# Spiritual Growth or Decline and Meaning-Making as Mediators of Anxiety and Satisfaction with Life During Religious Struggle

**DOI:** 10.1007/s10943-018-0598-y

**Published:** 2018-03-14

**Authors:** Beata Zarzycka, Pawel Zietek

**Affiliations:** 1 Institute of Psychology, The John Paul II Catholic University of Lublin, Al. Racławickie 14, 20-950 Lublin, Poland; 2 Department of Orthopaedics and Traumatology of Pomeranian Medical University, SPSK1, ul. Unii Lubelskiej 1, 71-252 Szczecin, Poland

**Keywords:** Religious struggle, Spiritual struggle, Meaning-making, Spiritual transformation, Mediation

## Abstract

A number of studies have demonstrated links between spiritual struggles and health problems. As yet, however, only a few studies have investigated what makes religious struggle a source of mental problems or a source of well-being. We determined whether spiritual growth, spiritual decline, and meaning-making mediated the relationship between religious struggle and anxiety and satisfaction with life. Of the 180 respondents, 92 were women, and mean (SD) age was 24 (8.2) years. Each respondent completed the Religious and Spiritual Struggles Scale, the Meaning-Making Scale, the Spiritual Transformation Scale, the State-Trait Anxiety Inventory, and the Satisfaction with Life Scale. Religious struggle correlated positively with anxiety and negatively with satisfaction with life. Spiritual growth mediated the relationship between moral and demonic struggle with satisfaction with life, and spiritual decline mediated the relationship between demonic, moral, and interpersonal struggle with anxiety. Finally, meaning-making mediated the relationship between religious doubt and satisfaction with life.

## Introduction

### Religious Struggle

For many people, religion is an important part of life. They turn to religion to receive consolation, to bond with others, to provide meaning to life experiences (Park 2005a, b), and to cope with adversity (Pargament 1997, 2007). Religion is also a source of social support, positive mental states, and a predictor of health (Park and Slattery 2013). Over the past decades, various academic researchers have established the beneficial effects of religiosity on personal adaptation and somatic and mental health (see for reviews George et al. 2002; Hill and Pargament 2003; Koenig et al. 2001; Larson and Larson 2003).

However, thinking about God does not necessarily bring only comfort and consolation. When people think that negative events in their lives are unfair or beyond their control (Kushner 1981), they can blame God for them and feel confused, distrust, or anger toward their God (Exline et al. 2011). Some people feel hurt by other believers, e.g., those with different beliefs or when they witness hypocrisy by clergy (Krause et al. 2000). There are also individuals who try to live in accordance with their religious beliefs but who cannot live up to the rules imposed by that religion and, as a result, feel guilty and are afraid that God will not forgive their failings. Such experiences generate strains and are a source of internal struggle (Wilt et al. 2016).

The notion of “religious and spiritual struggle” refers to these forms of distress or conflicts in the religious or spiritual realm (Zinnbauer et al. 1999). In psychology, “religious struggle” is defined from two theoretical perspectives. The first is the concept of religious coping, which defines struggle as a specific, negative form of religious coping elicited by various life stressors (Pargament et al. 2000). The second perspective defines struggle as a dispositional category; that is, a generalized tendency to experience strains related to religious beliefs and experience and not as a reaction related to religious coping (Exline 2013; Zarzycka 2017).

A religious or spiritual struggle can occur in several domains. The struggle may refer directly to God or be expressed as negative emotions or conflicts related to beliefs about God. It may manifest as doubts about religion, a sense of guilt toward God, losing one’s sense of meaning about life, or interpersonal conflicts related to religion. Struggle may also refer to spiritual forces of evil, including a belief that people are subject to the effects and attacks of evil spirits (Exline 2013; Exline et al. 2014; Exline and Rose 2013).

### Outcomes of Religious Struggle

Most studies show that religious and spiritual struggle is related to weak personal adjustment and poorer mental health. A meta-analysis of 49 studies (13,512 respondents) reported positive but small correlations between religious struggle and anxiety, depression, negative mood, a sense of guilt, and social dysfunctions (Ano and Vasconcelles 2005). Similar relationships were reported in a meta-analysis of 147 studies (98,975 respondents) (Smith et al. 2003). Escape through religious activity as a reaction to difficulties, as well as blaming God for the difficulties, correlated highly with depression.

Although negative health indicators are unquestionably conditioned by religious struggle (Ano and Vasconcelles 2005; Exline and Rose 2013; Smith et al. 2003), the issue how this conditioning happens has been understudied (Exline 2013). Nevertheless, some studies have identified variables that moderate or mediate relationships between religious struggle and health indicators. For example, the relationship between struggle and psychological distress is stronger in people living alone than in those in marriages (Ellison and Lee 2010), in younger people than in older people (Ellison and Lee 2010; Krause et al. 1999), and in the sick and abused than in healthy controls (Pargament et al. 2006). Moreover, studies confirmed that religious struggle mediates the relationship between stress and health: in people with few problems, religious guilt and a negative opinion about God mitigate negative mental health symptoms but are escalated when the stress perceived is high (Zarzycka 2017). Identification with a specific church or tradition also moderates dependencies between religious struggle and health symptoms (Ellison et al. 2013). These results indicate varied, often non-intuitive functions of religious struggle in various domains of mental life and thus should be explored further.

Although most studies suggest that religious struggle worsens health (Ano and Vasconcelles 2005; Exline and Rose 2013; Smith et al. 2003), some researchers believe that religious struggle may be beneficial (e.g., Desai 2006; Pargament et al. 2006). For example, studies on adults in churches near Oklahoma City shortly after the 1995 terrorist attack took place revealed that struggle correlates positively with indicators of post-traumatic growth (Pargament et al. 1998a). Some psychologists and some religious groups believe that individual growth may happen through suffering and that crisis is not a tragedy but a transition point important in individual development (e.g., Erikson 1968).

However, an intriguing question remains: what makes religious struggle a source of serious somatic or mental problems or be a source of well-being? So far, only few researchers have assessed the ways in which religious struggle might improve functioning. Holcomb and Nonneman (2004) confirmed that crises experienced by students (being exposed to people with different thinking styles, emotional crises, e.g., being unceremoniously dumped by a fiancée close to the wedding date or experiencing the death of a friend or family member) lead to a more complex and more reflective faith. Still, spiritual growth did not happen automatically. It was preconditioned by balancing the challenge with the support received for that challenge. Lack of adequate challenge resulted in stagnation, whereas lack of optimum social support increased the risk of losing faith (cf. Bryant and Astin 2008). Three factors may determine the relationship between struggle and growth: the intensity of the struggle, religious involvement, and the ability to cope with difficult situations (Pargament et al. 2006). People experience higher growth with more serious struggles. People who had lost their sight to blindness experienced even higher growth in terms of psychosocial functioning if they evaluated their loss higher meaning (Brennan 2002). The extent to which religion is integrated with the lifestyle is important in predicting the results of religious struggle. In case of high integration, religious struggles have negative consequences initially, but, as the time goes by, they lead to positive results (Kooistra and Pargament 1999).

The possibility that religious and spiritual struggle might lead to growth under some conditions is a compelling but difficult question, one that has received little attention to date. Complex and sometimes contradictory findings on this topic point to the need for more research to clarify the conditions under which struggles lead to growth versus decline (Pargament et al. 2006).

### Research Problem and Hypotheses

We sought to determine whether the relationship between religious struggle and anxiety and satisfaction with life was mediated by spiritual growth, spiritual decline, or meaning-making. The belief that religious struggles worsen mental health is well established (see for review Exline 2013; Exline and Rose 2013; Zarzycka 2017). However, the mechanisms responsible for these processes are less defined (e.g., Ellison et al. 2013; Ellison et al. 2010; Wortmann et al. 2012; Zarzycka 2017). The ways in which religious struggle may strengthen health and mental well-being are even less well understood. We sought to determine how religious struggle affects anxiety and satisfaction with life and whether these effects were sometimes positive. Specifically, we hypothesized that, through spiritual growth and the ability to make meaning of struggle, people would experience more satisfaction with life and less anxiety.

## Methods

### Respondents and Procedure

Of the 180 (92 women) respondents, the mean (SD) age was 24 (8.2) years. Most respondents were single and had at least a secondary school degree; all were Roman Catholic (Table 1). Respondents rated themselves above the midpoint on the six-point scale assessing level of religiosity [mean (SD), 4.29 (1.19)] and stress perceived [mean (SD), 3.13 (1.27)].

**Table 1:** Demographic characteristics of 180 respondents in a study assessing whether spiritual growth, spiritual decline, or meaning-making mediated the relationship between religious struggle and anxiety and satisfaction with life

Characteristic	*n*	%
Sex		
Female	92	51.1
Male	88	48.9
Education		
Elementary	2	1.1
Secondary	152	84.4
Higher professional	26	14.5
Marital status		
Singe	164	91.1
Married	16	8.9
Place of residence		
Village	77	42.8
City or town < 200,000 people	60	33.3
City > 200,000 people	43	23.9
Total	180	100

The study was conducted by the students of the third year of psychology at the Catholic University of Lublin as a part of a class titled, *Analysis and Interpretation of Empirical Data in Social Psychology*. Respondents were recruited through the Internet; the snowball method was applied. The researchers sent an invitation to the research to their friends by email; then these people forwarded this invitation to their friends. The inclusion criterion was experiencing a difficult situation in the previous 6 months, which triggered references to God. For their involvement in the study, the students were awarded credit points.

### Measurements

Respondents completed the 26-item Religious and Spiritual Struggle Scale, which assesses six domains of religious struggle: *Divine* (negative emotion centered on beliefs about God or a perceived relationship with God), *Demonic* (concern that the devil or evil spirits are attacking an individual or causing negative events), *Interpersonal* (concern about negative experiences with religious people or institutions), *Moral* (wrestling with attempts to follow moral principles; worry or guilt about perceived offenses by the self), *Religious Doubt* (feeling troubled by doubts or questions about one’s spiritual beliefs), and *Ultimate Meaning* (concern about not perceiving deep meaning in one’s life) (Exline et al. 2014; Zarzycka et al. in press). Respondents were asked to focus on the most stressful event they had faced in the past 6 months. The scale instruction included this prompt: “Recall a difficult event or situation which you experienced within the last 6 months and which induced references to God in you, e.g. you prayed to God, asked God for help, expressed your regret towards God, were angry at God, etc. Recall your thoughts and emotions towards God which you experienced at that time.” Then they completed the survey. Response options were from 1 (*not at all/does not apply*) to 5 (*a great deal*). The full scale and subscales were scored by averaging across items.

The Meaning-Making Scale measures respondents’ attempts to make meaning of stressful experiences (Abraham and Stein 2015). The scale was translated into Polish by the students. Theoretical conceptualizations and empirical research suggest that in attempting to understand one’s adverse experiences, individuals engage in cognitive processing, reappraisal, and emotional processing. Thus, the Meaning-Making process contains cognitive and emotional components (Abraham and Stein 2015). The Meaning-Making Scale combines subscales from two established measures to capture cognitive (Carver 1997) and emotional (Stanton et al. 2000) aspects of meaning-making. Respondents reported how often they used each meaning-making strategy to deal with their stressful situation. Responses options were from 1 (*not at all*) to 5 (*very often*). The Meaning-Making Scale makes it possible to receive a general result and results in two subscales: emotional processing (4 items) and positive reframing (3 items). In the present study, only the general result was considered and was calculated as the arithmetic mean of answers to all items.

The Spiritual Transformation Scale consists of 40 items measuring the intensity of spiritual changes across four domains: world view, life goals, relationships, and sense of self (Cole et al. 2008). It consists of two subscales: spiritual growth (29 items) and spiritual decline (11 items). The Spiritual Transformation Scale was translated into Polish by the students. Respondents were instructed to “Think about the experiences below and say which of them accompanied you when you struggled with the indicated difficult situation or were its result.” They then completed the 40 items using a scale from 1 (*strongly disagree*) to 7 (*strongly agree*).

Anxiety was measured using the State-Trait Personality Inventory. The inventory indicates the intensity of state anxiety (a temporary condition experienced in specific situations) and trait anxiety (a general tendency to perceive situations as threatening) (Spielberger et al. 1970). It consists of 40 items, 20 for trait anxiety and 20 for state anxiety. We used only the state anxiety items. Respondents were asked to indicate how they felt when they experienced the difficult situation they indicated earlier (e.g., I was tense; I was worried; I felt calm; I felt secure). Responses indicated intensity of feeling on a 1-to-4 scale, from 1 (*not at all*) to 4 (*very much so*).

The five-item Satisfaction with Life Scale is scored from 1 (*strongly disagree*) to 7 (*strongly agree*) (Diener et al. 1985). The instruction was to assess how much the respondents were satisfied with their life when they were experiencing a difficult situation.

We also collected data on age, sex, marital status, place of residence, and religious affiliation.

Data were collected through a web survey. Providing answers to a total of 98 questions from the five surveys generally required 30 min.

### Statistical Methods

We assessed the correlation among the key constructs of religious struggle, anxiety, satisfaction with life, meaning-making, and spiritual growth and decline. To this end, zero-order correlations were performed between the Meaning-Making Scale, Spiritual Transformation Scale subscales, State-Trait Anxiety Inventory, and Satisfaction with Life Scale.

**Fig. 1:**
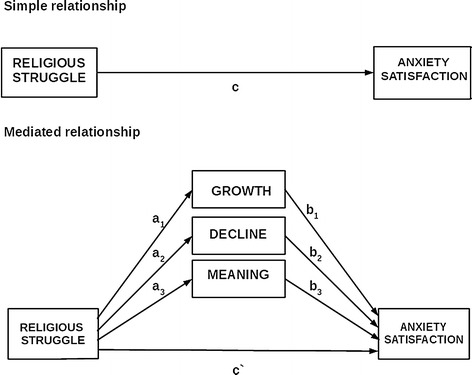
Our conceptual model of how spiritual growth, spiritual decline, and meaning-making might mediate the effect of religious struggle on anxiety and satisfaction with life. *c*—total effect of predictor on outcome without the mediator in the model; *c*′—direct effect of predictor on outcome while controlling for the mediator; *a*_1_*, a*_2_*, a*_3_—effect of the predictor on the mediator; *b*_1_*, b*_2_*, b*_3_—effect of the mediator on the outcome

In a regression model, the religious struggle subscales (divine, demonic, moral, ultimate meaning, interpersonal, and doubt) were examined for their relationship to anxiety and satisfaction with life. Spiritual growth, decline, and meaning-making were tested as mediators in these relationships. Figure 1 shows the conceptual mediation model.

Mediation, Moderation, and Conditional analyses were performed using PROCESS (Hayes 2013), a regression-based path-analytic framework and estimates the indirect effect and bias-corrected 95% confidence intervals (95% CI). An indirect effect is considered significant when the CI does not include zero. All analyses were based on 5000 bootstrapping samples. Bootstrapping is a nonparametric resampling procedure and, as such, it does not violate the assumption of normality (Serbic and Pincus 2017). Bootstrapping confidence intervals are presented for the indirect effects.

Data were analyzed with the SPSS statistical software program, version 24.

## Results

We first tested for missing observations and outliers. Respondents leaving more than 10% of the 98 questions blank were dropped (*n* = 5). The assumptions of normality were tested using the Kolmogorov–Smirnov test.

In all dimensions of the Religious Struggle Scale, the results were below 3 indicating low struggle (Table 2). The average score in spiritual growth and decline was below 4 (on a 1-to-7 scale) which shows a low level of spiritual transformation. In meaning-making the result was above 3 indicating a high score. Satisfaction with life was below 4 and state anxiety above 2, which demonstrates a low level of life satisfaction and a high level of anxiety. Men and women differed significantly in the degree of anxiety (female: *M* = 3.07, *SD* = .47; male: *M* = 2.72, *SD* = .54; *t*(173) = 4.58; *p* < .001) and spiritual growth (female: *M* = 3.84, *SD* = 1.43; male: *M* = 3.20, *SD* = 1.57; *t*(173) = 2.82; *p* = .05). To check the normality of the data, the coefficients of skewness, kurtosis, and the Kolmogorov–Smirnov test with Lilliefors correction were calculated for each construct. The coefficients of skewness and the K–S normality test indicate that the mean scores in the Religious Struggle Scale as well as in spiritual decline are slightly positively skewed, whereas the mean scores in other subscales are slightly negatively skewed. Most of the coefficient of skewness is less than 1, and the skewness is not strong enough and can be ignored. The values of kurtosis are also within the acceptable range. Cronbach alpha values for the subscales were either good or excellent (Table 2).

**Table 2:** Distribution of scores on variables in a study determining whether spiritual growth, spiritual decline, or meaning-making mediate the effects of religious struggle on anxiety and satisfaction with life among 180 respondents

Variable	*M* (*SD*)	Skewness	Kurtosis	Kolmogorov–Smirnov		Cronbach’s α
				Statistic	*p*	
Domains of religious struggle						
Divine	1.68 (.86)	1.61	2.48	.22	< .001	.89
Demonic	1.67 (.95)	1.44	1.31	.28	< .001	.92
Moral	2.31 (.99)	.41	− .66	.12	< .001	.79
Ultimate meaning	2.31 (1.18)	.57	− .76	.16	< .001	.91
Interpersonal	1.98 (.94)	.98	.23	.15	< .001	.83
Doubt	2.52 (1.13)	.27	− 1.03	.10	< .001	.87
Effect mediators						
Spiritual growth	3.52 (1.53)	− .02	− 1.08	.08	.01	.98
Spiritual decline	2.58 (1.29)	.64	− .56	.14	< .001	.92
Meaning-making	3.25 (.84)	− .52	.24	.09	.001	.81
Primary study outcomes						
State anxiety	2.89 (.53)	− .47	.25	.08	.009	.92
Satisfaction with life	3.57 (1.29)	− .22	− .72	.07	.021	.85

### Correlation Analyses

Pearson bivariate correlations were calculated for all variables examined in the regression model. The correlations between religious struggle and spiritual decline were all positive (Table 3). Spiritual growth correlated positively with demonic and moral struggle. The correlations between religious struggle and anxiety were also all positive (save for demonic struggle), whereas relationships between religious struggle and satisfaction with life were all negative (save for interpersonal struggle). Supplemental regression analyses revealed that religious struggle accounts for 6% of the variance in anxiety (*R*^2^ = .06, *p* = .02) and 14% of the variance in satisfaction with life (*R*^2^ = .14, *p* < .001).

**Table 3:** Correlations among dimensions of Religious and Spiritual Struggles Scale, Meaning-Making Scale, Spiritual Transformation Scale, Anxiety and Satisfaction with Life Scale

Variable	Variable number									
	1	2	3	4	5	6	7	8	9	10
1 Divine	–									
2 Demonic	.30***	–								
3 Moral	.39***	.54***	–							
4 Ultimate	.39***	.21***	.40***	–						
5 Interpersonal	.28***	.22**	.39***	.27***	–					
6 Doubt	.32***	.22**	.46***	.52***	.37***	–				
7 Meaning-Making	− .07	.04	.10	− .02	.07	.22**	–			
8 Growth	− .06	.27***	.26**	− .08	.02	.12	.37***	–		
9 Decline	.40***	.17*	.20**	.36***	.25**	.41***	.16*	.13	–	
10 Anxiety	.16*	.08	.20**	.24**	.19*	.23**	− .06	.02	.16*	–
11 Satisfaction	− .33***	− .19*	− .20**	− .32***	− .04	− .24**	.15*	.13	− .14	− .43***

*p < .05; ** *p* < .01; *** *p* < .001

### Mediation Analysis

The intermediary result of religious struggle on anxiety and satisfaction with life was significant (Table 4).

**Table 4:** Outcomes of mediation analyses from religious struggle to anxiety and satisfaction with life assessing indirect effects of spiritual growth, decline, and meaning-making

	Models without mediator		Models with mediator					Bootstrap results for indirect effects	
	B		B					95% CI	
	*R* ^2^	c	*R* ^2^	c′	a	b	ab	Lower	Upper
Struggle—Mediator—Anxiety									
DEM-Decline-Anxiety	< .01	.04	.04	.03	.23*	.06*	.02	.0005	.0490
MOR-Decline-Anxiety	.04**	.11**	.06*	.10*	.26**	.06*	.02	.0007	.0426
INTER-Decline-Anxiety	.04**	.11*	.06*	.10*	.33**	.05	.02	.0005	.0518
Struggle—Mediator—Satisfaction									
MOR-Growth-Satisfaction	.04**	− 1.29*	.11***	− 1.48**	.42**	.68*	.28	.0044	.7110
DEM-Growth-Satisfaction	.04*	− 1.28*	.04**	− 1.46**	.43***	.68*	.29	.0205	.7824
DEM-Decline-Satisfaction	.04*	− 1.28*	.11***	− 1.46**	.21*	− .72*	− .15	− .5154	− .0012
INTER-Decline-Satisfaction	< .01	− .28	.06*	− .07	.34***	− .85*	− .29	− .7091	− .0563
Doubt-Meaning-Satisfaction	.06**	− 1.37**	.12***	− 1.51**	.17**	1.41*	.23	.0304	.6159

DEM—demonic; MOR—moral; INTER—interpersonal; *B*—unstandardized regression weight; *c*—total effect of predictor on outcome without the mediator in the model; *c*′—direct effect of predictor on outcome while controlling for the mediator; *a*—effect of the predictor on the mediator; *b*—effect of the mediator on the outcome; *ab*—indirect effect of predictor on outcome through the mediator; *R*^2^—amount of variance explained by the model; *CI*—confidence intervals

*p < .05; **p < .01; ***p < .001

### Effect of Mediation on Anxiety

The indirect effect of demonic, moral, and interpersonal struggle was significant mediated by spiritual decline on anxiety. In two analyses, spiritual decline significantly increased the impact of moral and interpersonal struggle on anxiety. Spiritual decline also mediated the impact of demonic struggle on anxiety when compared to the regression model without the mediator (insignificant effect; Table 4).

### Effect of Mediation on Satisfaction with Life

Moral and demonic struggle, through spiritual growth, had a significant indirect effect on satisfaction with life. In two analyses, both moral and demonic struggle affected negatively on satisfaction with life (models without mediators), while, through spiritual growth, moral and demonic struggle increased satisfaction with life. Two analyses indicated that there was a significant indirect effect of demonic and interpersonal struggle, through spiritual decline on satisfaction of life: Spiritual decline increased the negative impact of demonic and interpersonal struggle on satisfaction with life. Finally, there was a significant indirect effect of doubt, through meaning-making on satisfaction of life. Religious doubt impacts negatively on satisfaction with life (model without mediators), whereas, through meaning-making, religious doubt increased satisfaction with life (Table 4).

## Discussion

We determined the function of spiritual growth, spiritual decline, and meaning-making in mediating the relationship between religious struggle and anxiety and satisfaction with life. That religious struggle can impair mental health is well established (Ellison et al. 2010; Exline 2013; Wilt et al. 2016), but its potentially positive functions have not been studied to the same extent. Thus, what factors determine whether religious struggle leads to psychological distress or well-being are unknown. We attempted to answer this question by studying people experiencing religious struggles during a difficult life situation to determine whether spiritual growth, spiritual decline, or meaning-making might predict the level of anxiety and satisfaction with life. Specifically, we hypothesized that, through spiritual growth and meaning-making in a difficult situation, religious struggle would reduce anxiety and increase satisfaction with life. Similarly, we also predicted that, through spiritual decline, religious struggles would increase anxiety and reduce satisfaction with life.

Correlational findings provided initial information about the relationships between religious struggle, spiritual growth and decline, meaning-making, anxiety and satisfaction with life. Generally, and as expected, religious struggle was related to anxiety and satisfaction with life. The correlations between religious struggle and anxiety were all positive (excluding demonic struggle), whereas relationships between religious struggle and satisfaction with life were all negative (except for interpersonal struggle). The negative consequences of religious struggle for mental health have been reported by others (e.g., Edmondson et al. 2008; Ellison and Lee 2010; Ellison et al. 2010).

Next, we checked whether spiritual growth, spiritual decline, and meaning-making mediated the relationship between religious struggle and anxiety and satisfaction with life. The hypothesis was that people who experience increased satisfaction with life and reduced anxiety are those who try to understand their struggle and whose struggle is a source of positive changes in world view, relationships, and their goals or sense of self (spiritual growth). However, when struggle leads to negative changes in the individual world view, relationships with others, or life goals (spiritual decline), anxiety increases and satisfaction with life decreases.

We found that the mediation effect was significant for four domains of struggle: demonic, moral, interpersonal, and religious doubt. In case of demonic and moral struggle, spiritual growth and spiritual decline were significant mediators. As we hypothesized, through spiritual growth, demonic and moral struggles can increase satisfaction with life, whereas spiritual decline can increase anxiety. In the relationship between interpersonal struggle and anxiety and life satisfaction, spiritual decline was significant.

We conclude that the effect of moral struggle on anxiety and satisfaction with life depends on the way moral strains are treated. When confronted with moral flaws and deficits in personality (religious struggle), if people observe in themselves positive changes in self-perception, perceiving the world and others, it increases their satisfaction with life. However, moral conflicts that lead to negative changes in self-perception and in perceiving the world generate anxiety. Some psychological theories (e.g., Erikson’s Theory of Psychosocial Development or Kohlberg’s Theory of Moral Development) focus on this function of moral conflicts; that conflict is a transition phase that can lead both to regression and to maturity and higher quality of life (e.g., Erikson 1968; Kohlberg 1976).

We observed similar mechanisms for demonic struggle: through spiritual decline demonic struggle increases anxiety and reduces satisfaction with life, whereas through spiritual growth the opposite was true. The fact that demonic struggle increases spiritual decline and thus generates anxiety and reduces satisfaction with life seems to be intuitive. Many people think that supernatural evil forces are active in the contemporary world and therefore they assign responsibility for suffering to these forces. Some studies confirm this view. For example, in some samples of Pentecostals, demonic interactions were one of the reasons given for mental dysfunctions (Harley 2007, Hartog and Gow 2005, za: Exline 2013). Other studies have found that some divorcees believe that the devil contributed to the divorce; that either they or their husband or wife were influenced by evil forces (Krumrei et al. (2011). This belief was correlated with anxiety and difficulties in adjustment (Pargament et al. 2000). The belief that one is influenced by the devil and assigning causation to the devil may lead to negative changes in perceiving the world and one’s self and, as a result, to anxiety and low satisfaction with life.

According to our study, demonic struggle may be also a source of positive change and lead to spiritual growth. Studies on the function of believing in the devil may be helpful in understanding this result. Many people believe strongly that the devil coexists with God and are thus less likely to blame God for their suffering (Beck and Taylor 2008) and so have little religious doubt (Exline et al. 2014). Therefore, the ability to assign negative events to evil forces may make it easier for people to keep their faith by keeping a positive image of self and God, decreasing anxiety and increasing satisfaction with life.

Spiritual decline significantly mediated the relationship between interpersonal struggle and anxiety and satisfaction with life. Interpersonal struggles increase anxiety and decrease satisfaction with life because they foster negative self-perceptions and negative assessments of one’s life goals. This finding is consistent with the idea that religion offers individuals several ways to maintain and enhance self-esteem, including unconditional positive regard, conditional positive regard, and opportunities for spiritual growth and development (Spilka et al. 1985). When religious beliefs are contested by others, the values of the religious person are challenged, which generates anxiety and reduces satisfaction with life (cf. Nielsen and Fultz 1995; Pargament et al. 1998b).

Religious doubt is an important part of believing (Hunsberger et al. 1996; Hunsberger et al. 2002; Kooistra and Pargament 1999). It is difficult to imagine a deeply religious person who does not have doubts about his or her religious beliefs. For example, Tillich (1957, p. 57) argued that “doubt is not the opposite of faith; it is an element of faith.” Similar views are expressed in the classic work of Allport (1950, p. 73), who maintained that, “the mature religious sentiment is ordinarily fashioned in the workshop of doubt.” However, studies on the relationship between religious doubt with mental and physical health are inconsistent. Although many confirm the negative effect of religious doubt on health, others have found no statistically significant relationship between doubt and psychological distress (Hunsberger et al. 2002), and still others have confirmed positive relationships (Exline et al. 2014; Krause and Wulff 2004). In the face of such inconsistent data, it is reasonable to ask about the mechanisms by which religious doubt might affect health (Krause and Wulff 2004). Our findings suggest that meaning-making regulates the impact of religious doubt on satisfaction with life: the better the ability to make meaning of religious doubt, the more benefits it brings through satisfaction with life. This finding is consistent with the idea that religious doubts and questioning might be part of a healthy process and suggests that meaning-making regulates the impact of religious doubt on satisfaction with life, as suggested by studies on quest approaches to religion (Batson and Schoenrade 1991; Exline et al. 2014).

### Strengths and Limitations of the Study

The basic limitation of the study is its cross-sectional design, which excludes any conclusions about cause–effect. The direction of interpretation applied in this study is based on theoretical assumptions. Longitudinal studies are needed to assess functions of religious struggles in terms of health and well-being. The study was based on individuals’ self-reports, and thus, the response bias could not be controlled. It is possible that the results reflect a social desirability bias. However, this possibility may be tempered somewhat by the fact that respondents completed the measures anonymously and they were asked about positive and negative outcomes. Arguably, if they were attempting to present themselves in a positive light, they would not have endorsed negative outcomes. Regardless, in the future, studies should incorporate scales assessing for social desirability, controlling for it if necessary. We also assumed that translating the survey instruments into Polish did not affect their performance characteristics.

## Conclusions

Meaning-making is significant in assessing relationships of religious doubt with well-being: satisfaction with life increases through the ability to make meaning of religious doubt. Moral and demonic struggle may both foster (through spiritual growth) and worsen (through spiritual decline) well-being; interpersonal struggle (through spiritual decline) contributes to worsening well-being, whereas religious doubt increases satisfaction with life (through meaning-making).
